# Antioxidant Activity of Natural Phenols and Derived Hydroxylated Biphenyls

**DOI:** 10.3390/molecules28062646

**Published:** 2023-03-14

**Authors:** Kristina Kostić, Jasmina Brborić, Giovanna Delogu, Milena R. Simić, Stevan Samardžić, Zoran Maksimović, Maria Antonietta Dettori, Davide Fabbri, Jelena Kotur-Stevuljević, Luciano Saso

**Affiliations:** 1 Department of Medical Biochemistry, Faculty of Pharmacy, University of Belgrade, Vojvode Stepe 450, 11221 Belgrade, Serbia; kostickristina77@gmail.com (K.K.);; 2 Department of Pharmaceutical Chemistry, Faculty of Pharmacy, University of Belgrade, Vojvode Stepe 450, 11221 Belgrade, Serbia; jbrboric@pharmacy.bg.ac.rs; 3 Sassari Unit, Institute of Biomolecular Chemistry of CNR, Traversa La Crucca 3, 07100 Sassari, Italy; giovanna.delogu@icb.cnr (G.D.); mariaantonietta.dettori@cnr.it (M.A.D.);; 4 Department of Organic Chemistry, Faculty of Pharmacy, University of Belgrade, Vojvode Stepe 450, 11221 Belgrade, Serbia; 5 Department of Pharmacognosy, Faculty of Pharmacy, University of Belgrade, Vojvode Stepe 450, 11221 Belgrade, Serbia; stevan.samardzic@pharmacy.bg.ac.rs (S.S.); zmaksim1@pharmacy.bg.ac.rs (Z.M.); 6 Department of Physiology and Pharmacology “Vittorio Erspamer”, Sapienza University of Rome, Piazzale Aldo Moro 5, 00185 Rome, Italy; luciano.saso@uniroma1.it

**Keywords:** phenolic compounds, oxidative stress, antioxidants, biological matrix, human serum

## Abstract

A comparative in vitro study of the antioxidant potential of natural phenols (zingerone, curcumin, raspberry ketone, magnolol) and their synthesized derivatives was performed. The antioxidant efficiency was evaluated in blood serum obtained from healthy individuals, by means of spectrophotometry, before and after the addition of pro-oxidant *tert*-butyl hydroperoxide (TBH). Moreover, the antioxidant effect of an equimolar mixture of curcumin and zingerone was investigated. Interpretation of our results reveals that in the blood serum of healthy individuals curcumin (C1), raspberry ketone (RK1), magnolol (M1) and synthesized derivative of zingerone (Z2) demonstrate remarkable antioxidant effects (*p* < 0.05). However, in the state of TBH-induced excessive oxidative stress natural magnolol and synthesized derivatives C1, Z1 and RK1 show powerful antioxidant activity and thus can be further investigated to obtain information about their metabolic transformations and their potential influence at the cellular level. Results obtained from measurements in an equimolar mixture of zingerone and curcumin indicate synergism (*p* < 0.05) between the two compounds. This combination is especially successful due to the fast and efficient neutralization of added pro-oxidant TBH. The commercial availability of turmeric and ginger and their frequent combined use in diet suggest ideas for further broader utilization of the beneficial synergistic effect of their phenolic components.

## 1. Introduction

### 1.1. Oxidative Stress and Natural Phenols as Antioxidants

In 1985, Sies was the first to use the term “oxidative stress” to describe an imbalance between pro-oxidant molecules (free radicals) and antioxidants in favour of the former, resulting in the disruption of redox signalling and molecular damage [1]. Free radicals are unstable atoms, atomic groups or molecules that have at least one unpaired electron, which makes them highly reactive, and their activity leads to the creation of a vicious circle of unwanted chemical processes. Excessive free radicals cause structural and functional modifications of macromolecules—DNA, proteins and lipids [2].

It is well known that oxidative stress underlies the pathogenesis of many leading causes of death [3,4,5]. Increased oxidative stress has been noted as a common sign in various diseases, during the earliest, as well as the advanced stage of disease development. Disruption of a fine balance between the presence of ROS and antioxidants in a healthy individual can eventually result in the complete evolvement of disease, where oxidative stress continues to prevail [3,4,5]. This shows the importance of the preventive and/or therapeutic application of antioxidants that will combat oxidative stress.

Antioxidants are substances with the ability to counteract the harmful effects of oxidants. Due to their usual insufficient production by the body, they frequently need to be supplemented by external sources [6].

Secondary metabolites of plants contribute to the functioning of plant organisms in which they are formed, and a considerable number of these compounds show pharmacological activity. Among the main groups of secondary metabolites of plants, phenols stand out with their wide range of benefits for the plant and other organisms. It is proven that they have a protective function for the plant. This function consists of preventing the infection of tissues with bacteria, fungi or viruses (phytoalexin function), protecting against an overdose of ultraviolet radiation, excessive transpiration or some other unfavourable environmental factors [7]. This spectrum of functions that secondary metabolites with phenolic structure express is a direct result of the significant metabolic reactivity of the phenolic group [8].

**Figure 1:**
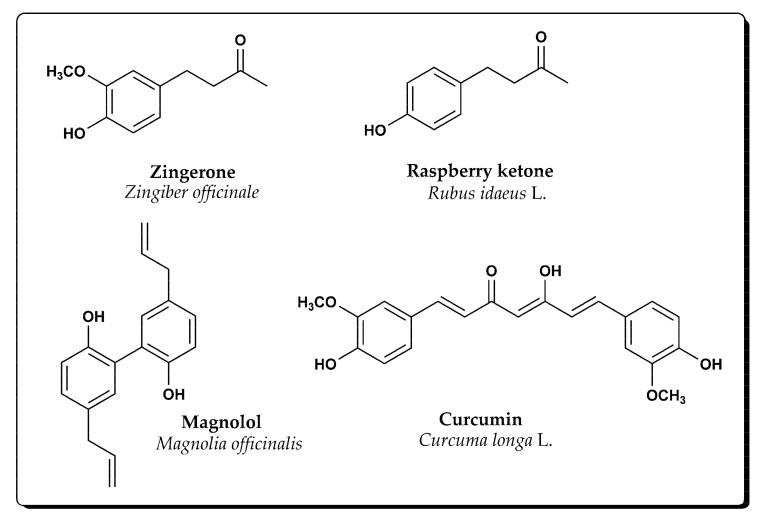
Chemical structures of some natural phenols.

Some known natural compounds of the phenolic type, among many others, are zingerone, curcumin, raspberry ketone and magnolol (Figure 1).

*Zingerone* [4-(4-hydroxy-3-methoxyphenyl)butan-2-one] is an active constituent isolated from dried or heat-treated ginger (*Zingiber officinale* Roscoe, family Zingiberaceae) [9]. Due to its distinct aroma, the ginger rhizome is often used in cooking. Zingerone has potent pharmacological properties as anti-inflammatory, antidiabetic [10] and antimicrobial activities [11].

*Curcumin* [(1*E*,4*Z*,6*E*)-5-hydroxy-1,7-bis(4-hydroxy-3-methoxyphenyl)hepta-1,4,6-trien-3-one] is an active component of the rhizome of the turmeric plant (*Curcuma longa* L., family Zingiberaceae) [12]. Originally from India, it is widely used as a culinary spice and natural colour. Curcumin has been largely used in traditional Chinese medicine thanks to its capacity to modulate the activity of many biological targets involved in mammalian physiology [13,14,15,16,17].

*Raspberry ketone* [4-(4-hydroxyphenyl)butan-2-one] is a significant aromatic component of raspberry (*Rubus idaeus* L., family Rosaceae) [18,19], used as a fragrant component in cosmetics and as an artificial flavour in food production [20]. At higher doses, raspberry ketone has shown the ability to reduce serum and liver lipids, thus reducing the risk of developing fatty liver and having a protective effect on hepatocytes [21].

*Magnolol* [5,5′-diallyl-[1,1′-biphenyl]-2,2′-diol] is a polyphenolic substance isolated from the bark of the traditional Chinese medicinal plant magnolia (*Magnolia officinalis*, family Magnoliaceae), which is used to relieve abdominal discomfort, cough and dyspnea [22]. Magnolol is often consumed in daily life, as it is a component of extract added to mints and gums, and recently, in cosmetics.

These natural phenolic compounds are known in both traditional and modern medicine due to their wide range of pharmacological effects. These healing effects are largely attributed to their antioxidant activity [17,21,23,24].

**Figure 2:**
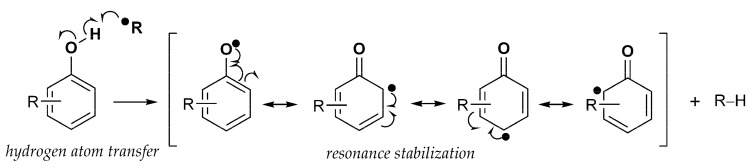
Formation and stabilization of substituted phenoxy radical.

Phenolic compounds are thought to best exert their protective antioxidant role by directly neutralizing free radicals. Such action involves a hydrogen atom transfer (HAT) mechanism and a single-electron transfer (SET) mechanism. In the first case, by transferring the hydrogen ion, the phenol molecule itself becomes a free radical (Figure 2). The stability and further reactivity of radicals thus formed directly depends on the structure of a molecule itself, i.e., possibilities of forming intramolecular hydrogen bonds and electronic delocalization [1]. 

However, evidence strongly suggests that mechanisms by which plant polyphenols exert their protective actions against diseases are not limited to their redox properties, but further extend to their ability to directly bind to target proteins (or peptides) [7]. In such a way, phenols can induce the inhibition of key enzymes, the modulation of cell receptors or transcription factors, as well as affect the signal transduction pathways in various ways. Among the most therapeutically relevant enzymes that undergo a change of activity in the presence of phenols are inflammatory ones such as cyclooxygenases (COXs) and lipooxygenases (LOXs), cytochrome P450 enzymes (CYPs), signal transduction kinases, xanthine oxidase, NADH-oxidase, thioredoxin reductase, adenosine deaminase, matrix metalloproteinases, telomerase, topoisomerases and methyl transferases, ATPase/ATP synthase, etc.

Numerous studies have been carried out in order to provide further insight into the physicochemical basics behind phenol–protein complexation (and precipitation) and how phenols binding to proteins could affect their biological activities, including their antioxidant action [8]. Some of the most relevant, although sometimes apparently conflicting, points drawn from these investigations can be summarized as follows:

- The nature and extent of the interactions between phenols and proteins strongly depend on the chemical structure and related physical properties of both phenols and proteins [7,8].
- Hydrophobic effects are usually the predominant cause of association, which is then further stabilized by hydrogen bonding [7,8].
- The conformational flexibility of phenolic biphenyl compounds constitutes an important determining factor of their ability to interact with proteins [7,8].

Taking into consideration the stated points, the antioxidant effect of phenolic compounds can scarcely be pinpointed, generalized or predicted. Additionally, this emphasizes why the need for new synthetic compounds is particularly important: not only in order to overcome a few features that many plant phenols are missing in order to become pharmaceutical drugs (most often poor bioavailability), but also to fuel the structure–activity relationship and in vivo interactions studies aimed at understanding the mechanisms of action of these classes of natural products. However, in a biological environment, phenolic compounds are able to counteract oxidative stress in other ways as well, such as by blocking the activity of enzymes responsible for generating O_2_^•−^, such as xanthine oxidase, protein kinase C, peroxidase and catalase [1]. Our study certainly could not answer all these questions about these compounds’ behaviour in living systems, and this warrants further investigation. 

### 1.2. Redox Properties of Hydroxylated Biphenyls

Hydroxylated biphenyls belong to the chemical class of polyphenolic compounds. Their characteristic pharmacophore includes two aromatic rings connected by a single C-C bond (aryl–aryl single bond). On several occasions, they have shown the ability to counteract oxidative stress even better than their corresponding monomers [25,26].

Hydroxylated biphenyls, as well as other phenolic substances, show a well-known tendency to interact with proteins (precipitating or modulating their activity), which directly affects their antioxidant potential. Analysis of NMR-derived binding data indicates that hydroxylated biphenyls have high affinity and specificity for a broad range of protein targets that could be utilised for the discovery and design of therapeutics [27]. The interaction between hydroxylated biphenyl and protein can be a direct consequence of conformational isomerism caused by hindered rotation around an aryl–aryl single bond, better known as *atropisomerism*. The macroscopic appearance of a particular atropisomer can be either due to hindered rotation around the axis by sufficiently large substituents, or because a diastereomeric orientation at a relatively unhindered axis is energetically preferred. The configurational stability of such biphenyl atropisomer can be estimated from its structure, in particular from the number of substituents next to the axis and their size [8,28]. The protein–hydroxylated biphenyl interaction would be enhanced with conformational flexible hydroxylated biphenyls whose structure can find effective interactions toward key amino acid residues.

The presence of two hydroxyl (phenolic) groups enables intramolecular hydrogen binding, which can significantly increase but also decrease the antioxidant effect. The formation of these bonds is very unpredictable and cannot be generalized. Presence of other substituents in the benzene ring, which also show the ability to form these bonds makes the situation even more complicated [29].

An intramolecular hydrogen bond is formed between nearby functional groups in both hydroxylated biphenyl and corresponding phenoxyl radical product, formed after the donation of an -H atom to neutralize a free radical. The hydrogen bond of the radical is stronger than that of the starting hydroxylated biphenyl, which reduces the energy required to break the bond between hydrogen and oxygen within the –OH group in hydroxylated biphenyl, i.e., increases the donor reactivity of hydroxylated biphenyl, to free radicals [29,30].

However, if a double hydrogen bond is formed between two phenolic –OH groups in a hydroxylated biphenyl molecule, a decrease in antioxidant reactivity is possible. Then, stabilized hydroxylated biphenyl is only partially balanced by the corresponding phenoxyl radical having only one hydrogen bond, which makes a free radical neutralization reaction by donating a hydrogen atom energetically unfavourable [29].

The aim of this study was to compare the antioxidant potential of several natural phenols (zingerone, curcumin, magnolol and raspberry ketone) and their synthetic derivatives, all with hydroxylated biphenyl structures. As a reaction medium, we used a human blood serum pool in order to make a natural environment which existed in systemic circulation for the tested compound, as well as considering the influence of protein binding on antioxidant activity.

## 2. Results

**Figure 3:**
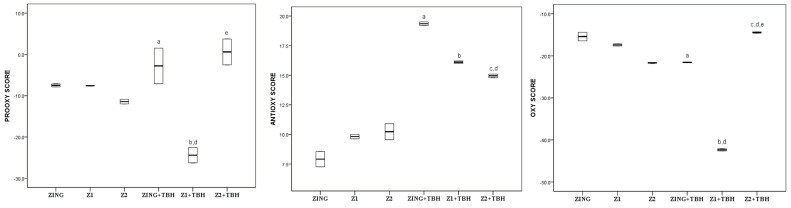
Values of pro-oxidative, antioxidative and oxidative scores of zingerone (ZING) and its two derivatives (Z1, Z2) in serum pool before and after the addition of TBH; Values are represented by median (horizontal line) and interquartile range (25–75th percentile (lower and upper edge, respectively)). a,b,c,d,e—significant difference (*p* < 0.05) compared to ZING, Z1, Z2, ZING + TBH, Z1 + TBH respectively.

Values of the pro-oxidative score, antioxidative score and oxidative score of zingerone and its two derivatives (Z1, Z2), before and after the addition of tert-butyl hydroperoxide, are graphically (boxplot) presented in Figure 3. 

The oxidative score of Z2 was lower than that of Z1 and zingerone, with statistical significance (*p* < 0.05). The addition of TBH decreased the oxidative score of all compounds significantly (*p* < 0.05). However, in the presence of TBH, Z1 + TBH demonstrated the lowest oxidative score (*p* < 0.05) compared to its precursor (ZING + TBH) and another derivative (Z2 + TBH). 

**Figure 4:**
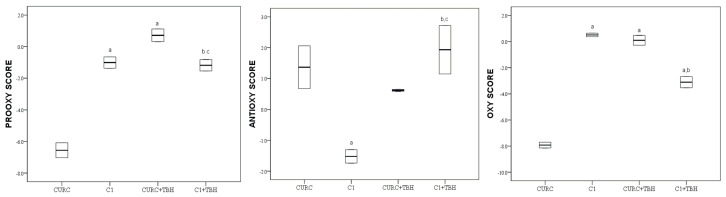
Values of pro-oxidative, antioxidative and oxidative scores of curcumin (CURC) and its derivative (C1) in serum pool before and after the addition of TBH; values are represented by median (horizontal line) and interquartile range (25–75th percentile (lower and upper edge, respectively)). a,b,c,—significant difference (*p* < 0.05)compared to CURC, C1, CURC + TBH, C1 + TBH, respectively.

Values of the pro-oxidative score, antioxidative score and oxidative score of curcumin (CURC) and its derivative (C1), before and after the addition of tert-butyl hydroperoxide (TBH), are graphically (boxplot) presented in Figure 4.

A significantly (*p* < 0.05) lower value of oxidative score of curcumin (CURC), in comparison to its derivative (C1), was observed. The addition of TBH changed the oxidative score of all compounds significantly (*p* < 0.05). After the addition of TBH, the oxidative score of CURC+TBH was significantly (*p* < 0.05) higher than that of C1 + TBH.

**Figure 5:**
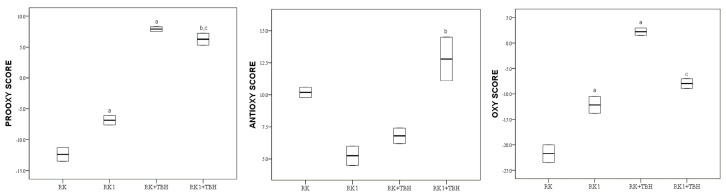
Values of pro-oxidative, antioxidative and oxidative scores of raspberry ketone (RK) and its dimer (RK1) in serum pool before and after the addition of TBH; values are represented by median (horizontal line) and interquartile range (25–75th percentile (lower and upper edge, respectively)). a,b,c,—significant difference (*p* < 0.05) compared to RK, RK1, RK + TBH, respectively.

The values of the pro-oxidative score, antioxidative score and oxidative score of raspberry ketone (RK) and its dimer (RK1), before and after the addition of tert-butyl hydroperoxide (TBH), are graphically (boxplot) presented in Figure 5.

The oxidative score of raspberry ketone (RK) was significantly lower (*p* < 0.05) than that of its dimer (RK1). TBH addition increased the oxidative score of RK significantly (*p* < 0.05), whereas the oxidative score of RK1 did not change with significance (*p* > 0.05). In the presence of TBH, RK1 + TBH obtained a significantly lower value of oxy score compared to its precursor (RK + TBH). 

**Figure 6:**
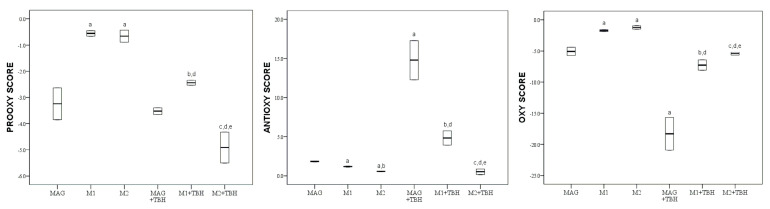
Values of pro-oxidative, antioxidative and oxidative scores of magnolol (MAG) and its two derivatives (M1, M2) in serum pool before and after the addition of TBH; values are represented by median (horizontal line) and interquartile range (25–75th percentile (lower and upper edge, respectively)). a,b,c,d,e—significant difference (*p* < 0.05)compared to MAG, M1, M2, MAG + TBH, M1 + TBH, respectively.

The values of the pro-oxidative score, antioxidative score and oxidative score of magnolol (MAG) and its two derivatives (M1, M2), before and after the addition of tert-butyl hydroperoxide, are graphically (boxplot) presented in Figure 6.

Magnolol showed a significantly (*p* < 0.05) lower oxidative score compared to both of its derivatives (M1, M2). The addition of TBH decreased the oxidative score of all compounds significantly (*p* < 0.05). Even in the presence of TBH, magnolol (MAG + TBH) maintained a statistically significant (*p* < 0.05) lower oxidative score compared to both derivatives (M1 + TBH, M2 + TBH). However, in such conditions, M1 + TBH showed statistically (*p* < 0.05) lower oxidative scores than M2 + TBH.

**Figure 7:**
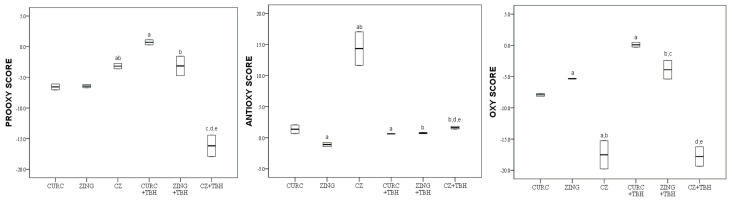
Values of pro-oxidative, antioxidative and oxidative scores of curcumin (CURC), zingerone (ZING) and an equimolar mixture of curcumin and zingerone (CZ) in serum pool before and after the addition of TBH; Values are represented by median (horizontal line) and interquartile range (25–75th percentile (lower and upper edge, respectively)). a,b,c,d,e—significant difference (*p* < 0.05) compared to CURC, ZING, CZ, CURC + TBH, ZING + TBH, respectively.

The values of the pro-oxidative score, antioxidative score and oxidative score of curcumin (CURC), zingerone (ZING) and an equimolar mixture of curcumin and zingerone (CZ), before and after the addition of TBH, are graphically (boxplot) presented in Figure 7.

The equimolar mixture of zingerone and curcumin (CZ) in the serum pool resulted in a significantly (*p* < 0.05) lower value of oxidative score compared to both CURC and ZING separately. With the addition of TBH oxidative scores of curcumin (CURC) and zingerone (ZING) increased significantly (*p* < 0.05). However, the oxidative score of CZ remained almost the same. In the presence of TBH, CZ + TBH preserved a significantly (*p* < 0.05) lower value of oxidative score compared to both CURC + TBH and ZING + TBH separately.

## 3. Discussion

### 3.1. Phenols as Antioxidants

The basis of antioxidant activity of all tested compounds in this investigation is a direct neutralization of free radicals, by virtue of a highly reactive phenolic group. The high reactivity of phenolic compounds can be understood as an advantage, which is a prerequisite for therapeutic application, but there are some concerns regarding their unpredictable behaviour in the biological medium. The overall antioxidant effect of these compounds in circulation depends on the presence of substituents in the aromatic ring that can activate intramolecular interactions and interactions with other components of the biological medium in such a way that the antioxidant capacity of the compound is effective. Proper substituents in the aromatic ring provide bioavailability of the compound that can reach sensitive comparts of the cell.

Using a comprehensive parameter of oxidative stress—oxidative score, calculated by means of Z score statistics, several different potential mechanisms of antioxidant activity of tested substances were included, as well as the fact that its antioxidant activity could be modified by binding to plasma proteins.

A major limitation of studies on phenolic compounds is their poor solubility, low absorption and bioavailability and high metabolism rate after oral administration. The suggested solution is based on these components’ encapsulation which could increase their bioavailability [31]. An experimental model of our research is performed in such a way that we hypothesized this limitation bypassing and analysing the final effect at the target site of action—i.e., circulation. Precisely, we assumed that polyphenolic compounds analyzed in this current study reach systemic circulation, which enables us to estimate its interaction with biomolecules existing in serum, even ex vivo, after the blood drawing procedure and serum sample separation from the whole blood sample.

### 3.2. Antioxidative Properties of Tested Compounds

Many studies declare zingerone as a potent antioxidant, and Rajan et al. even placed it ahead of ascorbic acid, in antioxidant activity [23]. Kancheva et al. showed a strong antioxidative activity of the zingerone’s dimer with phenolic –OH groups in the ortho position to the single C-C bond (Z1) [32]. Our results confirmed that conclusion as we now demonstrate an even more pronounced antioxidant effect of zingerone dimer with phenolic groups in meta position to the single C-C bond (Z2), compared to both zingerone and its dimer with phenolic –OH groups in ortho position to the single C-C bond (Z1) (Figure 3). Both zingerone derivatives (Z1, Z2), structurally hydroxylated biphenyls, have an electron-donating ortho-methoxy group that increases the ability for -H abstraction from phenolic –OH group and stabilizes the phenoxyl radical generated, i.e., increases the antioxidant potential. Additionally, as in the zingerone structure (Figure 1), both Z1 and Z2 possess a saturated side chain. Likely, the saturated side chain in the para position to the phenolic –OH group, exerts an electron-donating function to the aromatic group favouring the generation and stabilization of the phenoxyl radical [32]. Differently from Z1, in Z2 the two phenolic –OH group cannot activate intramolecular H-bond, therefore, the abstraction of the proton from the phenol–OH group is favoured.

**Figure 8:**
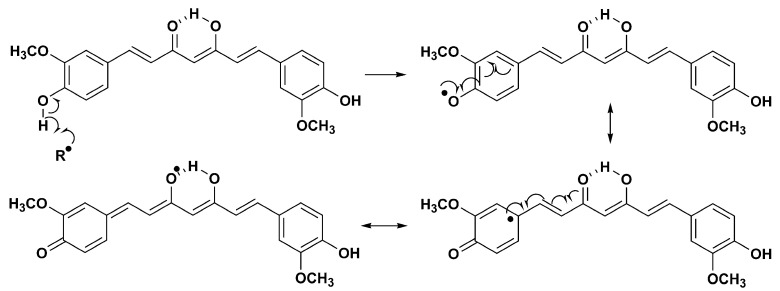
Phenolic group as radical source in the curcumin molecule.

A meta-analysis of randomized controlled trials (RCTs) conducted by Sahebkar et al., indicates a significant effect of curcumin supplementation on all investigated parameters of oxidative stress, in both healthy individuals and subjects diagnosed with certain illnesses (doses administered: from 80 to 1500 mg/day of curcumin; duration of supplementation ranged between 4 weeks and 8 weeks) [33]. Curcumin has an effect on free radicals through several different mechanisms. It can directly scavenge free radicals but also modulate the activity of glutathione, the enzymes catalase and superoxide dismutase, enzymes involved in free radicals neutralization [34]. Moreover, this natural phenolic compound inhibits ROS-generating enzymes such as lipoxygenase/cyclooxygenase and xanthine dehydrogenase/oxidase [35,36]. The radical scavenging activity of curcumin may be explained by the formation of stable radical species. Abstraction of hydrogen atoms can be performed from one or two phenolic groups (Figure 8). Stabilization of the resulting phenoxy radical takes place via the conjugated system [37,38]. The intramolecular hydrogen bond present in the enol form can have an effect on the stability of the radical.

A mechanism of autoxidation of curcumin was also proposed. In this reaction, the phenoxy radical of curcumin was transformed into a cyclopentandione derivative [38].

**Figure 9:**
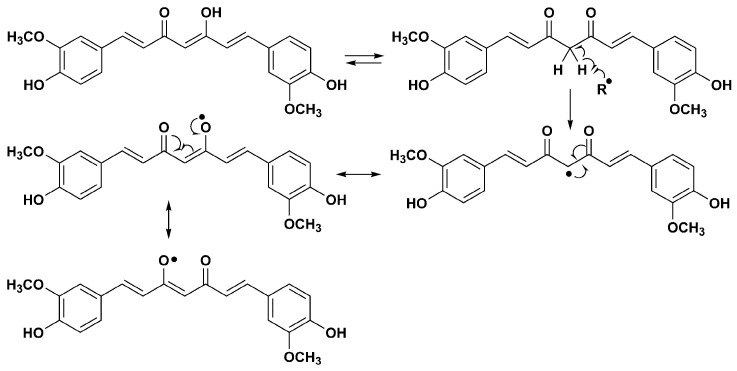
Methylene group as radical source in the curcumin molecule.

The radical can also be formed on the methylene group that exists in the keto tautomer of curcumin. In this case, resonance stabilization occurs via two keto groups (Figure 9).

Kancheva V. et al. concluded that the antioxidant activity of C1 was comparable to that of curcumin in lipid peroxidation [26]. However, in a biological medium, we demonstrated that curcumin and C1 are not comparable, with natural phenol showing stronger antioxidant potency (Figure 4). Curcumin is a compound where two substituted phenol moieties are linked by a seven-atom carbon chain in the para position to the hydroxyl group (Figure 1) while its derivative C1 is a 2,2′ hydroxylated biphenyl. The aliphatic array of seven carbon atoms connecting the two benzene rings in the curcumin structure is long enough that no intramolecular hydrogen bonds are formed between the phenolic groups and their independent antioxidant action is enabled. Unlike curcumin, its derivative C1 has a biphenyl structure which can initially lead to the formation of intramolecular hydrogen bonds that reduce reactivity to free radicals, as experimentally tested by Amorati et al. [29,30]. On the other hand, the unsaturated substituents in both aromatic rings of the C1 derivative have an electron-withdrawing effect and this structure does not have the stabilization capability present in curcumin molecules.

Examining the protective effect of raspberry ketone on nonalcoholic hepatitis in rats, Wang et al. found an increase in superoxide dismutase enzyme activity and a decrease in malondialdehyde content, indicating a protective effect of raspberry ketone from oxidative stress caused damages [21]. Our results confirm the effective antioxidant activity of raspberry ketone is even more significant than that of the synthesized dimer (RK1), as shown in Figure 5. RK1 is a hydroxylated biphenyl with –OH groups in the ortho position, and the formation of intramolecular hydrogen bonds can initially reduce the reactivity of the compounds to generate free radicals, according to the model proposed by Amorati [29]. Furthermore, it should be noted that no additional strong electron-donating group is present in the structure of RK1, so the ability of hydrogen atom abstraction from an OH-group is not increased [32].

**Figure 10:**
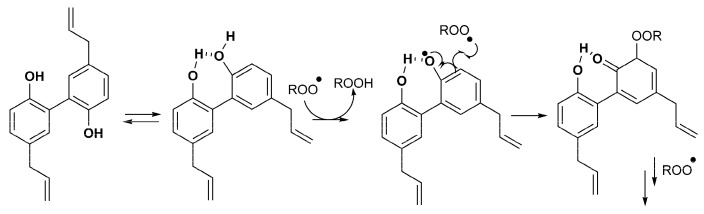
Radical scavenging activity of magnolol.

Magnolol is the only natural hydroxylated biphenyl examined. This molecule is known as a radical scavenger and the effect of two phenolic groups in a biphenyl structure is very important. Its M1 derivative has one methylated phenolic group and the M2 derivative has both methylated phenolic groups (methoxy groups). Our results are in agreement with the conclusion drawn by Baschieri—the protection of the phenyl–OH group by a methyl group reduces the reactivity of the compound against free radicals, therefore leaving MAG as the strongest antioxidant (Figure 6) [36]. Although intramolecular hydrogen bonds are also formed between both –OH groups of MAG and the methoxy group and the –OH group of M1, the dissociation energy of the bond is lower in the case of MAG, which makes it more reactive. Figure 10 represents the proposed mechanism of radical formation and radical scavenging activity of magnolol [30]. 

### 3.3. Antioxidative Properties of Tested Compound under Condition of Oxidative Stress

Tert-butyl hydroperoxide is an organic peroxide and an oxidizing and polymerizing agent usually used in experiments as a trigger of pro-oxidative reactions [39,40]. The addition of TBH to the blood serum pool of healthy individuals serves as a way of forming an experimental model of excessive oxidative stress, that can be found in the circulation of patients with a range of diseases, thus allowing us to make an assumption about the broad possible therapeutic antioxidant potential of examined compounds.

TBH addition to serum reaction mixture resulted in different effects on different compounds—decrease (Z2, CURC, RK) or increase (ZING, Z1, C1, MAG, M1, M2) the antioxidant activity of the compound. One possible explanation for the change in the behaviour of compounds lies in the complex interactions amongst phenol, protein and TBH. As previously described, phenolic compounds interact with proteins in serum which can result in an increase or decrease in antioxidant activity, depending on the overall mode of action by which certain compounds exert their antioxidant activity.

It has been proven that hydrophobic effects are usually the predominant cause of association, which is then further stabilized by hydrogen bonding, therefore making –OH groups of phenolic compounds occupied and unable to scavenge free radicals [7].

Zingerone and its derivative Z1 in the presence of TBH showed lower OS values compared to OS scores without TBH (Figure 3).

Even though Z2 showed a significant ex vivo antioxidant activity in the serum pool of healthy individuals, after the addition of TBH, Z1 demonstrated the ability to combat induced oxidative stress most efficiently. Interaction between the two phenolic groups in Z1 may decrease the antioxidant activity compared to the other derivative, Z2 in the vicinity of different biomolecules. At a higher concentration of pro-oxidants, the formation of a semiquinone structure in Z1 is possible, which would fight more effectively with reactive radical species.

In the curcumin group, the results of CURC and C1 also changed remarkably. In spite of the fact that CURC shows a notable ex vivo antioxidant activity in the blood serum of healthy individuals, its derivative C1 demonstrates a stronger antioxidant response in the conditions of TBH-induced oxidative stress (Figure 4). In this case, it is also possible that the oxidized dimerized form C1 becomes more capable of resisting oxidative stress, perhaps as a radical scavenger.

A similar situation occurs between RK and its dimer RK1, as the addition of TBH to RK1 exerts better antioxidant activity compared to its precursor, as shown in Figure 5. Despite the difference in the oxy score value of these two compounds, they still do not show antioxidant properties under conditions of oxidative stress. Ito et al. found that the enzymatic oxidation of raspberry ketone produces a cytotoxic quinone with pro-oxidative activity [41]. It is not excluded that the formation of an oxidized ketone is responsible for the increase in the oxy score.

However, in the magnolol series, the addition of TBH produced a significant increase in the antioxidative potential of the natural compound (MAG), which continued to be the most potent antioxidant in the group (Figure 6). It is acknowledged that hydroxylated biphenyls in virtue of their conformational flexible structure activate more and effective interactions with proteins in comparison to other aromatic structures. These results also confirm the mechanism that explains the antioxidant properties of magnolol. The resulting oxidized form has the ability to still react as a scavenger of free radicals (Figure 10). C1, RK1 and MAG could exert this effect [27].

### 3.4. Antioxidative Properties of Mixture of Natural Phenolic Compounds

Many natural phenols, components of food, beverages, dietary supplements and herbal drinks are called “nutraceuticals”, which emphasizes their beneficial effect on health [42]. The literature data indicate that the combination of two phenolic compounds may result in an additive, synergistic or antagonistic antioxidant effect [43,44]. In this study, we examined the antioxidant activity of an equimolar mixture of two natural phenols, zingerone and curcumin, isolated from ginger and turmeric, respectively, two culinary spices that are often combined in everyday use.

The result demonstrates that the equimolar mixture of zingerone and curcumin has a more prominent antioxidant effect in comparison with the corresponding individual components (Figure 7). The sum of individual oxy scores of zingerone and curcumin is higher than its mixture’s oxy score which suggests 0 for their synergistic action, i.e., ability to potentiate their favourable characteristics, having in mind that a larger oxy score value means worse antioxidant capability.

**Figure 11:**
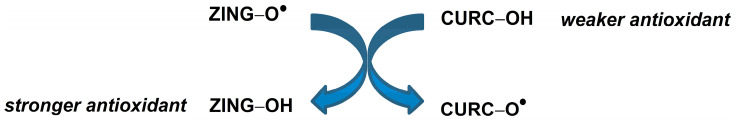
Interaction between two antioxidants.

The proposed mechanism of the synergistic effect is the regeneration of the stronger phenolic antioxidant by the weaker one in the pair, according to the model of α-tocopherol regeneration with ascorbic acid (Figure 11). The first step of this synergism involves the transfer of a hydrogen ion from a weaker antioxidant to a radical product of a stronger one, and the second involves the mutual neutralization of the radical products of both antioxidants [27,43,44,45]. Similarly, the weaker in the pair, curcumin, should undergo regeneration by the stronger one in the pair, that being zingerone.

Moreover, this combination is particularly distinguished by the fact that the addition of TBH to the reaction mixture does not change the total oxidative score, which indicates a rapid and efficient neutralization of pro-oxidants.

## 4. Material and Methods

### 4.1. General

NMR spectra were recorded on Bruker AscendTM 400 (Billerica, MA, USA) and Varian VXR 5000 spectrometers (Palo Alto, CA USA). Chemical shifts are given in ppm (δ); multiplicities are indicated by s (singlet), d (doublet), t (triplet), q (quartet), m (multiplet) or dd (doublet of doublets). Elemental analyses were performed using an elemental analyser Perkin-Elmer model 240 C (Walthan, MA USA). Melting points were determined on a Büchi 530 apparatus (Flawil, Switzerland) and are uncorrected. Flash chromatography was carried out with silica gel 60, 230–400 mesh (VWR, Radnor, AF, USA) eluting with an appropriate solution in the stated v:v proportions. Analytical thin layer chromatography (TLC) was performed with 0.25 mm thick silica gel plates (Polygram*^®^*Sil G/UV254, Macherey-Nagel (Oensingen. Switzerland).

Solvents and reagents, unless otherwise specified, were of analytical reagent grade purchased from Aldrich Chemie (Steinheimm, Germany) and Merck (Darmstadt, Germany).

An automatic analyzer ILab 300+ Instrumentation Laboratory (Milan, Italy) and ELISA plate reader BioTek (Winooski, VT, USA) were used for measuring the redox status parameters.

### 4.2. Chemistry

Phenolic compounds from plants (zingerone, magnolol and raspberry ketone) were commercially obtained, curcumin was isolated from turmeric, and their derivatives were synthesized. Curcumin was isolated by performing a procedure previously reported by Anderson et al. (2000) [46]. Briefly, a mixture of commercially available powdered turmeric rhizome (21.1 g) and dichloromethane (50 mL) was stirred and heated for one hour. Next, the mixture was vacuum filtered and the resulting filtrate was concentrated and triturated with hexane (20 mL). The residue was collected by vacuum filtration and dried. Its portion (0.5 g) was dissolved in a minimal amount of a mixture of dichloromethane and methanol (99:1, *v*/*v*) and loaded onto a silica gel column (30 g). Elution was performed with the same solvent mixture. The collected fractions were analyzed by analytical TLC using a mobile phase consisting of dichloromethane and methanol (97:3, *v*/*v*). The presence of three dominant components was observed. Fractions containing only the least polar of these components were combined and brought to dryness to give a yellow solid (109.9 mg) and were as follows: ^1^H NMR (DMSO-d_6_, 400 MHz) δ 3.85 (s, 6H), 6.07 (s, 1H), 6.76 (d, J = 15.8 Hz, 2H), 6.83 (d, J = 8.1 Hz, 2H), 7.16 (d, J = 8.1 Hz, 2H), 7.33 (s, 2H), 7.55 (d, J = 15.8 Hz, 2H), 9.65 (s, 2H); ^13^C NMR (DMSO-d_6_, 101 MHz) δ 56.18, 101.25, 111.86, 116.18, 121.58, 123.59, 126.82, 141.16, 148.47, 149.83, 183.68. The experimentally generated spectra agreed with the corresponding spectra of curcumin available in the literature [31], thus confirming the identity of the isolated compound.

#### Synthesized Compounds

Compounds **Z1** and **C1** were prepared according to procedures described by Marchiani et al. [47].

Magnolol derivatives **M1** and **M2** were synthesized following a slightly modified methodology of Sun et al. [48] changing solvent (from DMF to acetone) and base (from sodium carbonate to potassium carbonate) with improved yields. Compounds **Z2** and **RK1** were prepared according to Brboric et al. [49].

**C1** (3E,3′E)-4,4′-(6,6′-Dihydroxy-5,5′-dimethoxy-[1,1′-biphenyl]-3,3′-diyl)bis(but-3-en-2-one): to a stirred solution of dehydrodivanillin (2.00 g, 6.6 mmol) in acetone (50 mL) at room temperature and under N_2_, an aqueous solution (1 N) of LiOH (40 mL, 40.0 mmol) was added dropwise. The mixture was stirred at reflux for 12 h. Water and 10% HCl were cautiously added. The precipitate was filtered, washed with water and dried to afford **C1** as a yellow solid (83%): mp 242–243 °C. ^1^H NMR (CDCl_3_, 400 MHz) δ ppm 2.36 (s, 6H), 3.98 (s, 6H), 5.30 (bs, 2H), 6.60 (d, J = 16.0 Hz, 2H), 7.1 (d, J = 2.0 Hz, Ar, 2H), 7.14 (d, J = 2.0 Hz, Ar, 2H), 7.47 (d, J = 16.0 Hz, 2H); ^13^C NMR (CDCl_3_, 101 MHz) δ ppm 27.32, 56.22, 108.77, 123.57, 125.27, 125.44, 126.60, 143.51, 145.45, 147.36, 198.30; Anal. Calcd for C_22_H_22_O_6_: C, 69.10; H, 5.80; Found: C, 69.49; H, 5.74.

**Z1** (4,4′-(6,6′-Dihydroxy-5,5′-dimethoxy-[1,1′-biphenyl]-3,3′-diyl)bis(butan-2-one): to a solution of zingerone (1.58 g, 8.0 mmol) in dry dichloromethane (15 mL), a solution of methyl-tributylammonium permanganate (MTBAP) (1.30 g, 4.00 mmol) in dry dichloromethane (15 mL) was added dropwise at room temperature under N_2_. The solution was stirred at room temperature for 1 h and then washed with an aqueous solution of Na_2_S_2_O_5_ (50 mL) The organic layer was separated, washed with water, dried over Na_2_SO_4_ and evaporated to afford **Z1** as a white solid that was purified by flash chromatography using a 1:2 mixture of ethyl acetate:petroleum ether as eluent (65%): mp 85–86 °C. ^1^H NMR (CDCl_3_, 400 MHz) δ ppm 2.14 (s, 6H), 2.74–2.88 (series of m, 8 H), 3.90 (s, 6H), 6.01 (bs, 2H), 6.71 (d, J = 2.0 Hz, Ar, 2H), 6.73 (d, J = 2.0 Hz, Ar, 2H); ^13^C NMR (CDCl_3_, 101 MHz) δ ppm 29.50, 30.13, 45.46, 56.09, 110.64, 122.68, 124.38, 132.88, 140.90, 147.18, 208.11; Anal. Calcd for C_22_H_26_O_6_: C, 68.38; H, 6.78; Found: C, 69.49; H, 5.74. 

**Z2** (4,4′-(5,5′-Dihydroxy-4,4′-dimethoxy-[1,1′-biphenyl]-2,2′-diyl)bis(butan-2-one): to a solution of 4-(4-isopropoxy-3-methoxyphenyl)butan-2-one (0.21 g, 0.89 mmol) in dichloromethane (15 mL), a solution of molybdenum (V) chloride (0.48 g, 1.78 mmol) in dichloromethane (10 mL) was added at 0 °C and under N_2_. The mixture was stirred at 0 °C for 45 m. Water was cautiously added. The solution was extracted with dichloromethane and dried over Na_2_SO_4_. The crude product was purified by column chromatography using a 1:1 mixture of petroleum: acetone as eluent, to give **Z2** as a yellow oil (60%): 1H NMR (CDCl_3_, 400 MHz) δ ppm 1.99 (s, 6H), 2.60–2.73 (series of m, 8H), 3.87 (s, 6H), 6.62 (s, Ar, 2H), 6.71 (s, Ar, 2H); ^13^C NMR (CDCl_3_, 101 MHz) δ ppm 27.05, 29.87, 45.12, 55.91, 111.34, 116.18, 130.62, 132.97, 143.38, 145.81, 208.35; Anal. Calcd.for C_22_H_26_O_6_: C, 62.55; H, 5.14; Found: C, 62.54; H, 5.12.

**RK1** (4,4′-(6,6′-Dihydroxy-[1,1′-biphenyl]-3,3′-diyl)bis(butan-2-one): methyl-tributylammonium permanganate (MTBAP) (0.49 g, 1.5 mmol) in dry dichloromethane (15 mL) was added at room temperature, dropwise and under N_2_ to a solution of raspberry ketone (0.5 g, 3 mmol) in dry dichloromethane (20 mL). The reaction mixture was stirred at 20 °C for 1 h and then was washed with an aqueous solution of Na_2_S_2_O_5_ (50 mL). The organic layer was separated, washed with water, dried over Na_2_SO_4_ and rotoevaporated to give crude **RK1** as a brown solid. Purification by flash chromatography using a 1:1 mixture of ethyl acetate:petroleum ether as eluent gave **RK1** as a white solid (46%): mp 83–84 °C. 1H NMR (CDCl_3_, 400 MHz) δ ppm 2.14 (s, 6H), 2.70–2.01 (series of m, 8H), 6.88 (d, J = 7.6 Hz, Ar, 2H), 7.10 (dd, J = 2.4, 7.6 Hz, Ar, 2H), 7.13 (d, J = 2.4 Hz, Ar, 2H); ^13^C NMR (CDCl_3_, 101 MHz) δ ppm 28.08, 30.15, 45.35, 116.96, 125.24, 129.21, 131.22, 133.63, 151.21, 209.51; Anal. Calcd. for C_20_H_22_O_4_: C, 73.60; H, 6.79; Found: C, 73.66; H, 6.74.

**M1** (5,5′-Diallyl-2′-methoxy-[1,1′-biphenyl]-2-ol) and **M2** (5,5′-diallyl-2,2′-dimethoxy-1,1′-biphenyl): magnolol (1 g, 3.75 mmol) was dissolved in acetone (20 mL), potassium carbonate (4 g, 28 mmol) and methyl iodide (3.2 g, 22.5 mmol) were added and the mixture was stirred for 5 h in an oil bath at 55 °C. The reaction was filtered and the solvent rotoevaporated. The residue was purified by flash chromatography using a 1:50 mixture of ethyl acetate:petroleum ether as eluent to obtain compounds **M1** and **M2** in 60% and 35% yield, respectively.

**M1**: yellow oil, ^1^H-NMR (CDCl_3_, 400 MHz) δ 3.37 (d, J = 6.4 Hz, 2H), 3.39 (d, J = 6.4 Hz, 2H), 3.88 (s, 3H), 5.04–5.12 (series of m, 4H), 5.93–6.02 (m, 2H), 6.22 (bs, 1H), 6.96 (d, J = 8.4, Ar, 1H), 6.98 (d, J = 8.4, Ar, 1H), 7.04 (d, J = 2.0, Ar, 1H), 7.12 (dd, J = 2.0, 8.4, Ar, 1H), 7.15 (d, J = 2.0, Ar, 1H), 7.22 (dd, J = 2.0, 8.4 Hz, Ar, 1H); ^13^C NMR (CDCl_3_, 101 MHz) δ 39.33, 39.45, 56.33, 111.60, 115.55, 115.89. 117.50, 126.22, 127.10, 129.17, 129.33, 131.22, 132.44, 132.61, 133.79, 137.42, 137.83, 152.04, 153.84; Anal. Calcd for C_19_H_20_O_2_: C, 81.40; H, 7.19; Found: C, 81.47; H, 7.22.

**M2**: m.p.: 44–45 °C, 1H-NMR (CDCl_3_, 400 MHz) δ ppm 3.39 (d, J = 6.4 Hz, 4H), 3.78 (s, 6H), 5.04–5.12 (series of m, 4H), 6.00 (m, 2H), 6.90 (d, J = 8.4, Ar, 2H), 7.06 (d, J = 2.0, Ar, 2H), 7.12 (dd, J = 2.0, 8.4, Ar, 2H); ^13^C NMR (CDCl_3_, 101 MHz) δ ppm 39.42, 55.88, 111.14, 115.50, 127.83, 128.43, 131.61, 131.76, 137.84, 155.49; Anal. Calcd for C_20_H_22_O_2_: C, 81.60; H, 7.53; Found: C, 81.67; H, 7.52. 

The NMR spectrum of compound **C1** confirms the geometry of the α,β-unsaturated ketone moieties. In fact, the large coupling constants (16 Hz) between olefinic protons indicate the exclusive presence of a trans isomer. Furthermore, the presence of only one set of aromatic and aliphatic signals confirms the formation of a dimeric structure with C_2_ symmetry. The synthetic procedure for **Z1** leads to the exclusive and selective formation of a C2 symmetric dimer with the bond between the two aromatic systems in ortho positions with respect to the hydroxyl substituents. This geometry is confirmed by the small coupling constant (2 Hz) between the aromatic protons at 6.71 and 6.73 ppm indicating an NMR meta interaction between the two aromatic protons. The NMR spectrum of compound **Z2** confirms the geometry of the C2 symmetric dimer in fact, the two aromatic protons present in each aromatic ring exhibit no coupling constant appearing as singlets at 6.62 and 6.71 ppm, suggesting a mutual para position. Moreover, unlike compound **Z1**, compound **Z2** has two hydroxyl substituents in the meta position with respect to the 1,1′ bond between the two aromatic rings. The structure of compound **RK1** was uniquely confirmed by comparison with the NMR literature data [50]. Compound **M1** is, among hydroxylated biphenyl structures reported in this study, the only one without C2 symmetry. This characteristic is evident from the analysis of the NMR spectra in which the presence of two sets of signals both relating to aromatic and aliphatic protons is highlighted.

**Figure 12:**
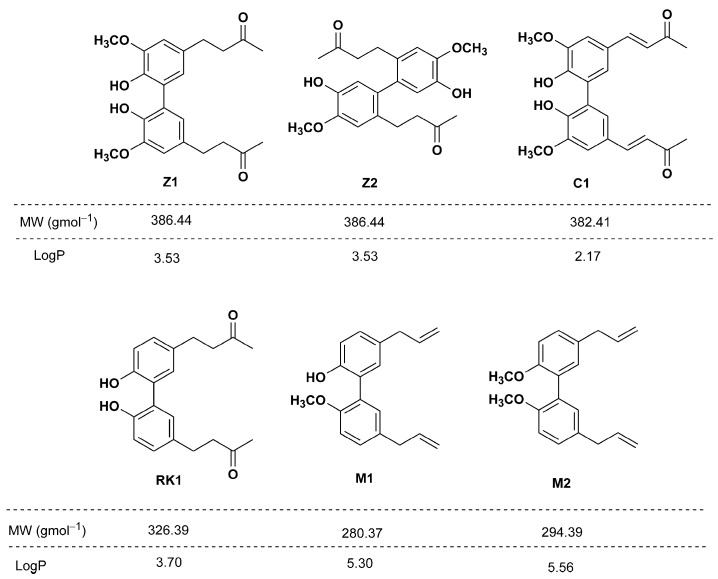
Structural formulas of tested compounds.

The structural formulas and lipophilicity of compounds **Z1**, **Z2**, **C1**, **RK1**, **M1** and **M2** were estimated by ChemBioDraw Ultra 13.0 software (CambridgeSoft) using the logarithm of the partition coefficient for n-octanol/water (LogP) and listed in Figure 12.

All examined compounds were soluble in dimethyl sulfoxide (DMSO) at a concentration of 15 mg/mL. All compounds were prepared and examined and compared at a concentration of 0.03 mol/L. An equimolar mixture of zingerone and curcumin contained 0.015 mol/L of each compound, respectively.

### 4.3. Evaluation of In Vitro Antioxidant Potential (Pro-oxidant/Antioxidant Activity) of the Compounds in Biological Matrix (Human Serum Pool) 

#### 4.3.1. Sample Collection

Healthy volunteers who had attended their regular medical check-ups at the Military Medical Academy in Belgrade had given approval that any serum remaining after biochemical analyses planned by physicians could be used for this study. Fifty samples whose basic biochemical parameters were within metabolite reference ranges were selected. After thorough mixing, the serum pool was aliquoted into 450 µL portions and frozen at −83 °C until analyses.

To 450 µL of serum, 50 µL of solution of each compound (at a concentration of 0.030 mol/L in DMSO) under investigation was added (this gives 500 µL in total) and then incubated at 37 °C for 2 h. As for the mixture of curcumin and zingerone, 25 µL of each compound was added (a total of 50 µL). The same procedure was implemented for the samples with a concomitant presence of tested substances and tert-butyl-hydroperoxide (TBH) (0.5 µL/mL solution in distillate water) as a pro-oxidant substance. All analyses were performed in triplicate.

#### 4.3.2. Total Oxidative Potency (TOP)

All oxidants in the sample (for example, H_2_O_2_ and lipid hydroperoxides) oxidise a ferro-orthodianisidin complex to a ferric ion in an acidic environment, in the presence of glycerol. The resulting ferric ion forms a coloured complex with xylene-orange. Colour intensity is measured spectrophotometrically (at 560 nm) and is proportional to the total content of oxidizing molecules in the sample [51,52] (Supplementary Materials, Table S1).

#### 4.3.3. Pro-oxidative–Antioxidative Balance (PAB)

PAB indicates a concomitant pro-oxidant load and antioxidative capacity of a particular organism. The assay determines the concentration of H_2_O_2_ in an antioxidative environment. The chromogen 3,3′,5,5′-tetramethylbenzidine (TMB) reacts with both H_2_O_2_ and antioxidants (including uric acid and other reducing species). The reaction between H_2_O_2_ and chromogen is catalysed by the enzyme peroxidase, resulting in the oxidation of TMB to produce an intense colour. In contrast, the reaction between uric acid and similar compounds with chromogen is not catalysed by peroxidase, and cation reduction causes discolouration. The colour generated in the reaction is proportional to the ratio of pro-oxidants and antioxidants. Absorbance was read at 450 nm after a 10 min incubation of the reaction medium at 37 °C [51,53] (Supplementary Materials, Table S1).

#### 4.3.4. Total Sulphydryl Groups (SHG)

Total sulphydryl groups in serum were determined by a modification of Ellman’s method, based on the formation of a yellow-coloured reaction product between 5,5′-dithio-bis(2-nitrobenzoic acid) (DTNB) and aliphatic thiol compounds in basic conditions (pH = 9.0). Absorbance was measured at 412 nm [51,54] (Supplementary Materials, Table S1).

#### 4.3.5. Total Antioxidant Capacity (TAC)

TAC was measured using the stable ABTS^▪+^ radical cation as a chromogen. ABTS is oxidised by H_2_O_2_ in acetate buffer; pH = 3.6 to a green-coloured ABTS^▪+^ radical cation. Antioxidants present in the sample make varying degrees of discolouration proportional to their concentration (the antioxidant potential of the sample). After incubation for 10 min at room temperature, absorbance was recorded at 600 nm [51] (Supplementary material, Table S1).

#### 4.3.6. Pro-oxidative Score, Antioxidative Score and Oxy Score

The database of obtained measurement results was formed in the program Microsoft Excel 2010. In the same program, the principle of Z score statistics was applied, on the basis of which the oxidative score of each tested compound is determined.

The oxidative score (oxy score) is the difference between the pro-oxidative score and the antioxidative score of the tested compound.

Pro-oxidative score (pro-oxy score) is the mean value of the Z score of measured pro-oxidant parameters—total oxidative potency (TOP) and pro-oxidant–antioxidant balance (PAB).

Antioxidant score (antioxy score) is the mean value of the Z score of measured antioxidant parameters—total antioxidant capacity (TAC) and total sulphydryl groups.

Z score is the difference between the sample parameter value and the mean value of a control serum (population mean) divided by the standard deviation (SD) of the control serum (population standard deviation).

The value of the oxidative score is inversely proportional to antioxidant protection, i.e., a lower value of the score indicates a stronger antioxidant activity of examined substance.

#### 4.3.7. Statistical Analysis

For further statistical analysis of the obtained results, the software package Statistics Package for the Social Sciences for Windows (SPSS, IBM Corporation, Chicago, Illinois, USA) ver. 18.0 was used. Results of all parameters were presented as numerical descriptive quantities—measurement of central tendencies (medians) and percentiles. The non-parametric tests—Mann–Whitney U test and Wilcoxon pair test were used for statistical analysis. The criteria for the existence of statistically significant differences are defined for the level of significance of 0.05, i.e., *p* < 0.05.

## 5. Conclusions

This study presents a comparative evaluation of the antioxidant properties of natural phenols and their synthetic derivatives with biphenyl motif performed in human serum which was obtained from healthy individuals. Natural curcumin, raspberry ketone and magnolol, as well as a synthetic dimer of zingerone (Z2) demonstrated remarkable antioxidant effects ex vivo in an environment containing relevant biomolecules such as proteins and lipids. In the state of TBH-induced excessive oxidative stress, natural magnolol and synthesized derivatives, C1, Z1 and RK1 showed also significant antioxidant capacity. Comparative analysis of obtained results indicated that there was no strict advantage of natural phenols over their synthetic biphenyl derivatives and vice versa and also reveal the potential of biphenyl derivatives of natural polyphenols to be the starting point for further development of agents with pronounced antioxidant properties.

Further studies of the antioxidant properties of a mixture of curcumin and zingerone confirmed their synergistic effect, which was suggested by a better profile of the mixture than that of the individual components. This combination was especially successful due to the fast and efficient neutralization of added pro-oxidant. Our results support the combined use of turmeric and ginger, as frequently seen in various diets, and promote further investigation of the antioxidant properties of these and related compounds.

Remarkable antioxidative properties of studied phenols may be influenced by an inadequate bioavailability due to their poor solubility but we believe that the advanced drug delivery approaches can resolve this problem and this remains to be addressed in our future studies.

## Data Availability

All the available data is contained in the manuscript.

## Supplementary Materials

The following supporting information can be downloaded at: https://www.mdpi.com/article/10.3390/molecules28062646/s1, Table S1: Redox status parameters concentration in serum sample.

## Sample Availability

Samples of the compounds **Z1**, **Z2**, **C1**, **RK1**, **M1** and **M2**, are available from the authors.
