# Supplementary material for: Antioxidant Activity of Natural Phenols and Derived Hydroxylated Biphenyls
Source: Molecules. 2023 Mar 14;28(6):2646. doi: 10.3390/molecules28062646 (PMC10053952; doi:10.3390/molecules28062646)
Supplement: Supplementary file 1 [file molecules-28-02646-s001.zip › molecules-2239821-supplementary.pdf]

molecules

MDPI

Supplementary Materials

Antioxidant Activity of Natural Phenols and Derived Hydroxylated Biphenyls

Kristina Kostić [1], Jasmina Brborić [2], Giovanna Delogu [3], Milena R. Simić [4,*], Stevan Samardžić [5], Zoran Maksimović [5], Maria Antonietta Dettori [3], Davide Fabbri [3], Jelena Kotur-Stevuljević [1] and Luciano Saso [6]

[1] Department of Medical Biochemistry, Faculty of Pharmacy, University of Belgrade, Vojvode Stepe 450, 11221 Belgrade, Serbia
[2] Department of Pharmaceutical Chemistry, Faculty of Pharmacy, University of Belgrade, Vojvode Stepe 450, 11221 Belgrade, Serbia
[3] Sassari Unit, Institute of Biomolecular Chemistry of CNR, Traversa La Crucca 3, 07100 Sassari, Italy
[4] Department of Organic Chemistry, Faculty of Pharmacy, University of Belgrade, Vojvode Stepe 450, 11221 Belgrade, Serbia
[5] Department of Pharmacognosy, Faculty of Pharmacy, University of Belgrade, Vojvode Stepe 450, 11221 Belgrade, Serbia
[6] Department of Physiology and Pharmacology "Vittorio Erspamer", Sapienza University of Rome, Piazzale Aldo Moro 5, 00185 Rome, Italy
* Correspondence: milena@pharmacy.bg.ac.rs

Table S1. Redox status parameters concentration in serum sample.

	PAB (U/L)	TOP (µmol/L)	SHG (mmol/L)	TAC (µmol/L)	PABtbh (U/L)	TOPtbh (µmol/L)	SHGtbh(mmol/L)	TAC tbh(µmol/L)
ZING	65.7 (67.6-63.8)	6.31 (7.84- 4.79)	0.31 (0.29- 0.32)	1097.48 (1090.75-1104.21)	92.53 (88.2-96.87)	17.1 (9.4-24.74)	0.33 (0.32-0.33)	1515.75 (1509.02-1522.48)
Z1	64.2 (64.8-63.7)	6.92 (7.01-6.82)	0.33 (0.32-0.33)	1179.21 (1181.13-1177.29)	31.6 (35.73-27.47)	11.78 (13.17-10.39)	0.17 (0.16-0.19)	1697.48 (1699.4-1695.56)
Z2	13.1 (13.2-12.9)	16.64 (11.73-21.55)	0.3 (0.28-0.32)	1264.79 (1261.90-1267.67)	107.97 (125.6-90.33)	12.98 (6.31-19.65)	0.16 (0.15-0.18)	1651.33 (1651.33-1651.33)
CUR C	138.93 (135.63-142.23)	25.9 (25.1-26.7)	0.55 (0.51-0.59)	1309.5 (1316-1303)	182.93 (187.43-178.43)	44.75 (44.2-45.3)	0.45 (0.53-0.38)	1359.5 (1331-1388)
C1	152.7 (148.9-156.5)	31.2 (29.6-32.8)	0.61 (0.53-0.69)	1337.5 (1335-1340)	160.1 (171.77-148.43)	34.6 (30.2-39)	0.41 (0.38-0.45)	1342 (1337-1347)
RK	13.47 (13.4-13.5)	15.53 (7.66-23.4)	0.32 (0.31-0.34)	1242.67 (1234.98-1250.37)	138.1 (128.4-147.8)	10.76 (6.04-15.48)	0.17 (0.17-0.17)	1011.9 (1130.17-893.63)
RK1	56 (55-57)	14.51 (9.88-19.14)	0.18 (0.16-0.2)	1235.94 (1234.98-1236.9)	131.6 (124.93-138.27)	5.39 (4.74-6.04)	0.16 (0.15-0.17)	1469.6 (1460.94-1478.25)
MAG	85.57 (86.27-84.87)	54.6 (58.49-50.71)	1.09 (1.15-1.03)	926.33 (898.44-954.21)	96.93 (96-97.87)	20.16 (20.57-19.74)	1.04 (1.06-1.02)	1398.44 (1574.4-1222.48)
M1	98.47 (97.2-99.73)	41.13 (46.18-36.08)	1.21 (1.24-1.19)	554.21 (527.29-581.13)	103.80 (103.67-103.93)	19.14 (18.17-20.11)	1.00 (0.86-1.15)	694.60 (630.17-759.02)
M2	102.57 (101.8-103.33)	27.19 (27.29-27.1)	1.16 (0.99-1.32)	208.06 (225.37-190.75)	95.23 (87-103.47)	4.97 (15.94-(-6.00))	1.04 (0.98-1.09)	389.79 (364.79-414.79)
CUR C	138.93 (135.63-142.23)	25.9 (25.1-26.7)	0.55 (0.51-0.59)	1309.5 (1316-1303)	182.93 (187.43-178.43)	44.75 (44.2-45.3)	0.45 (0.53-0.38)	1359.5 (1331-1388)
ZING	137.50 (141.77-133.23)	28.55 (26.5-30.6)	0.4 (0.4-0.39)	1345.5 (1354-1337)	177.93 (180.63-175.23)	40.45 (42.2-38.7)	0.32 (0.31-0.32)	1414 (1422-1406)
C+Z	167.97 (169.03-166.9)	26 (28.8-23.2)	1.05 (1.18-0.92)	1384 (1378-1390)	158.47 (158.1-158.83)	27.65 (27.3-28)	0.31 (0.3-0.33)	1466.5 (1460-1473)
